# *Staphylococcus aureus* Prostatic abscess: a clinical case report and a review of the literature

**DOI:** 10.1186/s12879-017-2605-4

**Published:** 2017-07-21

**Authors:** David E. Carroll, Ian Marr, G Khai Lin Huang, Deborah C. Holt, Steven Y. C. Tong, Craig S. Boutlis

**Affiliations:** 1 Department of Infectious Diseases, Royal Darwin Hospital, Darwin, Northern Territory Australia; 2 Territory Pathology, Northern Territory Department of Health, Royal Darwin Hospital, Darwin, Northern Territory Australia; 3 Menzies School of Health Research, Charles Darwin University, Casuarina, Northern Territory Australia; 4 Daisy Hill Hospital, Southern Health and Social Care Trust, Newry, Northern Ireland United Kingdom; 5 The Queen’s University of Belfast, Belfast, Northern Ireland United Kingdom; 6 Victorian Infectious Disease Service, The Royal Melbourne Hospital, Victoria, Australia; 7 Peter Doherty Institute for Infection and Immunity, The University of Melbourne, Victoria, Australia

**Keywords:** Prostatic abscess, *Staphylococcus aureus*, nmMRSA, MRSA, MSSA, Prostatic abscesses, PVL, ST5-MRSA

## Abstract

**Background:**

Prostatic abscess is a rare complication of acute bacterial prostatitis and is most commonly caused by *Enterobacteriaceae*. We report on a case of prostatic abscess caused by *Staphylococcus aureus* and conduct a review of the literature.

**Case presentation:**

We present a case of *S. aureus* prostatic abscess that was successfully treated with a combination of antibiotic and surgical therapy. The isolate was non–multidrug-resistant, methicillin-resistant *Staphylococcus aureus* and was genotyped as clonal complex 5, an emerging regional clone that is trimethoprim resistant and Panton-Valentine leukocidin positive. This current case report is the first to describe the use of clindamycin step-down therapy*.* A literature review identified a further 39 cases of *S. aureus* prostatic abscesses, of which 26 were methicillin resistant.

**Conclusions:**

*S. aureus* is an uncommon cause of prostatic abscess. Optimal management includes both antibiotic therapy and surgical drainage. Our use of clindamycin as step-down therapy was guided by its excellent prostatic penetration.

**Electronic supplementary material:**

The online version of this article (doi:10.1186/s12879-017-2605-4) contains supplementary material, which is available to authorized users.

## Background

Prostatic abscess is a rare complication of acute bacterial prostatitis, described in 0.5 to 2.5% of patients presenting with inflammatory prostatitis [1]. Patients with prostatic abscess typically present with difficulty urinating or with acute urinary retention. The most common causative pathogens include Enterobacteriaceae such as *Escherichia coli* [2], or in our local clinical context in northern Australia, *Burkholderia pseudomallei* [3]. *Staphylococcus aureus*, a Gram-positive coccus, is a common human pathogen, yet is a rarely reported cause of prostatic abscess [1].

We report here a case of prostatic abscess due to *S. aureus* in a man who presented to our institution and contextualise this with a literature review.

## Case presentation

The patient was a 53 year-old Indigenous Australian who presented to a remote regional hospital in northern Australia with acute onset urinary retention. His past history included type 2 diabetes mellitus, chronic hepatitis B infection and recurrent skin and soft tissue infections. He also had a history of long-term kava use.

An indwelling urinary catheter (IDC) was inserted and culture performed of the IDC urine specimen; however, the patient refused further treatment and self-discharged the same day (following removal of the IDC). At day 2, the initial urine culture revealed methicillin-resistant *S. aureus* (MRSA).

The patient was recalled and admitted to hospital. His urinary symptoms and associated suprapubic pain persisted. He had difficulty ambulating due to pain in his left thigh. A digital rectal examination revealed a firm prostate of normal size, without tenderness, and a tender diffuse swelling was noted in the medial left thigh. A beside ultrasound of the thigh revealed a hypoechoic lesion measuring 74x52x61 mm with an estimated volume of 123 mL. The patient had blood taken for culture, was then commenced on intravenous vancomycin and then transferred by air ambulance to our institution, where he was admitted for ongoing care.

Laboratory tests on admission showed a total leucocyte count of 12.7 × 10^9^/L (neutrophil count, 8.7 × 10^9^/L) and a CRP of 65 mg/L (reference range 0.0–5.0 mg/L). Serological testing for *B. pseudomallei* was negative at <1:20 using indirect haemagglutination. *B. pseudomallei* was not isolated from throat or rectal swabs after culture in Ashdown’s medium [4]. Urine culture again isolated a pure growth of MRSA, with reported antibiotic sensitivity to clindamycin but resistance to trimethroprim. This profile is consistent with non–multidrug-resistant, methicillin-resistant *S. aureus* (nmMRSA; isolates being defined phenotypically as those resistant to less than 3 non–beta lactam antibiotic classes [5]). An ulcer present on the right great toe was swabbed and was also culture-positive for nmMRSA with an identical antibiogram. Skin scrapings on this admission tested positive for *Sarcoptes scabei* and his clinical condition was consistent with a concomitant diagnosis of crusted (previously, Norwegian) scabies. He also had an ulcer on his right great toe.

A CT scan of the abdomen and pelvis showed multiple prostatic abscesses including a prominent expansive abscess within the left base and multiple loculations within the apex (Additional file 1: Fig. S1).

At day 2 post-transfer, screening blood cultures isolated nmMRSA with the same antibiogram as was found in urine on the first assessment. Vancomycin Etest® showed an MIC of 1.5 μg/mL.

This patient underwent immediate surgical drainage of the thigh abscess on day 1 following transfer and at day 5 underwent trans-rectal ultrasound guided drainage of the prostate, whereupon 5 mL of purulent fluid was aspirated from the superior margin of the left lobe. The prostate aspirate and operative samples from the thigh abscess cultured nmMRSA with the same resistance profile as previously; *B. pseudomallei* was not isolated. The collections of fluid in the right lobe of the prostate were unable to be drained because of their small size, anatomical location and thickness of the wall of the prostate.

A follow-up CT scan post-drainage of the prostatic abscess showed interval improvement in the loculated right prostatic abscess; however, the left-sided collection appeared unchanged with possible enlargement (Additional file 2: Fig. S2), measuring 21 × 35 mm compared with 19 × 21 mm prior to drainage. Following three days of IV antibiotic therapy and following surgical drainage of the abscess, surveillance blood cultures were negative. Transthoracic echocardiogram showed no evidence of endocarditis.

The patient was continued on intravenous vancomycin with addition of oral clindamycin because of its recognised prostatic penetration [6]. He declined further surgical drainage options. The patient improved clinically and received a total 4 weeks of intravenous vancomycin plus oral clindamycin therapy. Repeat imaging at the completion of IV therapy showed some residual abscess collection (not shown). Further surgical intervention was discussed with the patient and was declined. Repeat imaging performed 5 months later showed the prostatic abscess had completely resolved.

### Molecular microbiological genotype of disease

The *S. aureus* isolates recovered from the blood cultures and an additional isolate recovered from the prostate aspirate underwent molecular genotyping as previously described [7]. The isolates genotyped as Clonal Complex (CC) 5. Notably, *lukPV* (encoding for Panton-Valentine leucocidin [PVL]) was detected by PCR in both isolates.

## Literature review

### Methods

Two investigators independently and in duplicate conducted a review of the literature searching the MEDLINE and Cochrane databases for the terms “(prostate OR prostatic) AND aureus AND abscess*” from January 1946 through January 2017 inclusive to identify all relevant published literature. Non-MEDLINE indexed literature was also consulted and citation searching was undertaken on all results to identify further studies. Results were limited to peer-reviewed publications in English. Approval for this case report and study was granted by the Human Research Ethics Committee of the Northern Territory Department of Health and the Menzies School of Health Research (HREC number 2015–2457).

## Results

Thirty nine publications were identified. After screening the titles and abstracts, 21 publications met the inclusion criteria and were thus eligible for full-text review. An additional 14 papers were identified by reference screening for further citations that met inclusion criteria. The case presented in this study has been added to studies identified in the review.

These 35 full-text papers included a total of 39 patients with a diagnosis of *S. aureus* prostatic abscess. With our report of a further case, this led to a review of 40 cases. The summary of the literature search is displayed in Table 1.

**Table 1:** Summary of the current literature on *S. aureus* prostatic abscess

Ref	Age	Susceptibility	Presentation	Risk Factors	Bacteremia	Drainage	Antibiotic Used	Outcome
[8]	42	MRSA	Dysuria, urinary retention, abdominal pain	Urinary retention	No	Transrectal	Levofloxacin, metronidazole, fluconazole	Resolution
[14]	31	MRSA	Dysuria, abdominal pain	Immunosuppression, recent IDC, T2DM, liver transplant	Yes	No drainage.	Vancomycin, ceftriaxone, daptomycin	Resolution
[15]	20	MSSA + PVL	Dysuria	None	Yes	Transrectal	Ceftriaxone, amikacin, ciprofloxacin, rifampicin, ofloxacin, fosfomycin	Resolution
[16]	56	MRSA	Dysuria, polyuria, difficulty urinating	Diabetes mellitus	No	Radiology guided then TURP	Vancomycin	Resolution
[17]	47	MRSA	Diffuse myalgias, dry cough, anorexia, dyspnea, fever, and chills	Recent penile furuncle, history of hypospadias with urethral stricture	No	No drainage	Vancomycin, piperacillin/tazobactam	Death
[17]	31	MRSA	Left-sided pleuritic chest pain, fevers, rigors, and fatigue	Sickle beta	Yes	Transrectal and TURP	Vancomycin, piperacillin/tazobactam, daptomycin, TMP-SMX	Resolution
[18]	52	MRSA	Abdominal pain, urinary frequency	Diabetes mellitus	Yes	TURP	Vancomycin, TMP-SMX, rifampin	Resolution
[19]	47	MRSA	Dysuria, pyrexia, urinary frequency, rectal tenesmus, right flank pain	None	No	TURP	Vancomycin, piperacillin-tazobactam, linezolid.	Resolution
[19]	20	MRSA	Pyrexia, lower abdominal pain and perineal discomfort	IVDU, hepatitis C	No	Transurethral	Vancomycin, piperacillin-tazobactam, linezolid	Resolution
[20]	49	MRSA	Dysuria, urinary retention, pyrexia, scrotal pain and oedema, constipation	BPH	No	TURP	Vancomycin	Resolution
[21]	15	MRSA	Left buttock pain, constipation and difficulty ambulating	Previous bilateral lower extremity and back abscesses - grew MRSA	No	TURP	Clindamycin, piperacillin-tazobactam, then vancomycin, linezolid	Resolution
[22]	49	MRSA	Difficulty urinating, low back pain	Diabetes mellitus	Yes	TURP	Vancomycin, doxycycline	Resolution
[23]	65	MSSA	Dysuria, flank pain, and urinary retention, constipation, suprapubic pain	Diabetes mellitus	Yes	Transurethral drainage.	Ceftriaxone, ciprofloxacin	Resolution
[24]	45	MRSA	Dysuria, perineal discomfort, rectal tenesmus and intermittent pyrexia	Diabetes mellitus	Yes	Percutaneous, TURP	Vancomycin	Resolution
[25]	59	MRSA	Dysuria, urinary frequency, pyrexia, rectal and perineal pain	Diabetes mellitus, alcoholism	Yes	Percutaneous	Vancomycin, TMP-SMX, ciprofloxacin	Resolution
[26]	40	MRSA	Fever, urinary hesitancy, intermittent abdominal pain	HIV	Yes	Transperineal	Vancomycin, gentamicin	Resolution
[27]	50	MSSA	Urinary retention	Diabetes mellitus, alcoholism	No	No drainage	Ciprofloxacin	Resolution
[28]	51	MRSA	Dysuria, fever, suprapubic pain	HIV, sepsis, adult respiratory distress syndrome, and renal failure	Yes	Transurethral	Vancomycin, ciprofloxacin, TMP-SMX	Death
[29]	64	MRSA	Dysuria,fatigue, anorexia, and weight loss	Diabetes mellitus	Yes	Percutaneous	Vancomycin	Resolution
[30]	55	MRSA	Urinary hesitancy and rectal pain	BPH	Yes.	No Drainage	Vancomycin	Resolution
[31]	56	MRSA	Difficulty urinating, hematuria, fever, chills and low back pain	Diabetes mellitus, BPH, history of self-digital rectal examinations	Yes.	Percutaneous, TURP	Vancomycin, rifampin	Resolution
[32]	29	MRSA	Persistent pyrexia and superficial skin abscesses on his left calf. Worsening suprapubic and perineal pain	Straddle injury to urethra, urethroplasty, hepatitis C	No	Cope loop catheter placement	Vancomycin	Resolution
[33]	43	MRSA	Dysuria, hesitancy, slow stream, night sweats, flushing, and perineal pain	Intravenous drug abuse, hepatitis C	Yes	TURP	Vancomycin, nafcillin, TMP-SMX	Resolution
[34]	63	MRSA	Urinary retention, pyrexia	Recent scrotal drainage, T2DM	Yes	Percutaneous, TURP	Vancomycin	Unknown
[35]	59	SA [unknown susceptibility]	Difficulty passing urine	Recent salivary gland abscess treated with antibiotics	No	Transperineal, TURP	Ciprofloxacin, erythromycin	Resolution
[36]	35	SA	“clinical acute prostatitis”	HIV, bilateral renal abscesses	No	No drainage	“IV antibiotics”	Resolution
[37]	42	MSSA	Dysuria. urethral discharge, perineal pain	No	No	Transperineal	Oxytetracycline	Resolution
[38]	43	SA [unknown susceptibility]	Dysuria, haematuria, pyrexia, purulent discharge	Recent pelvic infection and pelvic surgery	No	Transurethral	Gentamicin, carbenicillin.	Resolution after relapse
[9]	54	MSSA	Perineal pain	Mycosis fungoides	No	Transperineal, TURP	Oxacillin, tobramycin	Resolution
[39]	53	MRSA	Urinary urgency, fatigue, chills, rigors, and fever	Diabetes mellitus, Obesity	Yes	No drainage	Vancomycin, TMP-SMX, rifampin	Resolution
[40]	50	MRSA + PVL	Abdominal pain, cough, fever, weight loss, malaise	Diabetes mellitus	Yes	CT guided drainage	Vancomycin, levofloxacin, daptomycin	Resolution
[41]	46	MRSA	Acute urinary retention, dysuria, constipation	Instrumentation	Yes	Transurethral	IV Antibiotics	Resolution
[42]	57	MRSA	Dysuria, haematuria, pyrexia	Diabetes Mellitus	Yes	TURP	Ceftriaxone, vancomycin	Resolution
[43]	67	SA[unknown susceptibility]	Acute urinary retention, perineal pain, dysuria, pyrexia	Diabetes mellitus	Yes	No drainage	Ciprofloxacin	Resolution
[44]	12	SA [unknown susceptibility]	Acute urinary retention, pyrexia	None	-	Transurethral	“anti-bacterials”	Resolution
[45]	69	SA [unknown susceptibility]	Pyrexia, lethargy and problems voiding	Diabetes mellitus	Yes	Transurethral	Ampicillin, oxacillin	Resolution
[46]	Newborn	MSSA	Urinary retention	No	No	No drainage	No therapy	Resolution
[46]	14 days	MSSA	Urinary retention, diarrhoea, bladder distension	No	No	Open drainage	No therapy	Resolution
[46]	3 weeks	MSSA	Abdominal distension	Hirschsprung’s disease	Yes	Open drainage	No therapy	Death
This case	52	nmMRSA + PVL	Urinary retention	Diabetes mellitus, crusted scabies, long term kava use	Yes	Transrectal	Vancomycin, clindamycin	Resolution

Legend: BPH [Benign Prostatic Hypertrophy]; CT [Computed Tomography]; HIV [human immunodeficiency virus]; IDC [Indwelling catheter]; IV [Intravenous]; PVL [Panton Valentine leucocidin]; SA [*Staphylococcus aureus*]; MSSA [Methicillin-sensitive *Staphylococcus aureus*]; nmMRSA [non–multidrug-resistant, methicillin-resistant *Staphylococcus aureus*]; MRSA [Methicillin-resistant *Staphylococcus aureus*]; T2DM [Type 2 Diabetes mellitus]; TMP-SMX [Trimethoprim/sulfamethoxazole]; TURP [transurethral resection of the prostate]

### Clinical presentation

The most common clinical presentations of prostatic abscess in this population were pain (abdominal or perineal) and pyrexia. This was followed by dysuria and urinary retention (Table 2). In our case, the presenting complaint was similar to those in previously published cases.

**Table 2:** Symptoms in patients with *S. aureus* prostatic abscess

Symptom	Total No. Patients (40)	% Of Patients
Pain/discomfort	21	52.5%
Pyrexia	20	50%
Dysuria	17	42.5%
Urinary retention	11	27.5%
Difficulty micturating	8	20%

MRSA was the dominant strain type causing prostatic abscess in the published literature, being identified in 26 (65%) cases of disease, including in the case that we report here. MSSA was identified as the causative strain in 8 cases. In 6 cases, the susceptibility of the causative bacterium was unreported. Genes encoding for PVL were identified in 3 cases, most notably in our case; however, there is little detail on how frequently the presence of *lukSF-PV* was sought in the existing literature.

### Risk factors for developing *S. aureus* prostatic abscess

Risk factors observed in this population of *S. aureus* associated prostatic abscesses include: diabetes mellitus, prior urethral instrumentation or catheterisation, prostatitis, and immunocompromise (Table 3).

**Table 3:** Risk Factors in patients with *S. aureus* prostatic abscess

Risk factor	Total No. Patients (40)	% Of Patients
Diabetes mellitus	17	42.5%
History of urological disease	5	12.5%
Recent surgery/instrumentation	5	12.5%
Immunosuppression (inc. HIV)	4	10%
Hepatitis C	3	7.5%

### Bacteraemia

23/40 (57.5%) cases in this report had positive blood culture results for *S. aureus* but due to variable quality of reporting in the literature, we are unable to determine if all cases had blood cultures taken*.* In addition one patient [8] had urinary cultures positive for *Candida albicans* and one patient [9] was co-infected with *E. coli*, *Bacteroides* and *Klebsiella pneumoniae*.

### Treatment

The majority of patients identified were treated with antibiotic therapy, most commonly with vancomycin. 22/40 (55%) patients were treated with antibiotic combinations, 15/40 (37.5%) were treated with mono-therapy and 3/40 (7.5%) with no antibiotic therapy (Table 4). In those patients with *S. aureus* bacteraemia (SAB), in addition to prostatic disease, vancomycin was again the most frequently used antibiotic, in keeping with international guidelines [10].

**Table 4:** Antibiotic Choice in patients with *S. aureus* prostatic abscess

Antibiotic	Total No. Patients [44]	% Patients
Vancomycin	24	60%
Trimethoprim/Sulfamethoxaxole	6	15%
Ciprofloxacin	7	17.5%
Piperacillin + Tazobactam	5	12.5%
Rifampin	4	10%
Cefriaxone	4	10%
Linezolid	3	7.5%
Daptomycin	3	7.5%

There is a paucity of evidence regarding the role of drainage in the treatment of prostatic abscess. In our review, 26/40 (65%) cases underwent drainage (Table 5). Several patients required drainage on more than one occasion. On each of those occasions, a trans-rectal or trans-perineal approach was followed by a transurethral approach to achieve successful drainage.

**Table 5:** Method of drainage in patients with *S. aureus* prostatic abscess

Drainage	Total No. Patients (40)	% Of Patients
Transurethral drainage	22	55%
Percutaneous drainage	8	20%
No drainage	8	20%
Transperineal drainage	5	12.5%
Transrectal drainage	4	10%

### Clinical outcome, microbiological outcome and relapse

In this review, 36/40 (90%) of cases identified resolved, one of which was following an initial relapse. Three cases died and the outcome in one case was unknown.

## Discussion

*S. aureus* is a major human pathogen that causes a wide range of clinical infections and there is a heavy burden of staphylococcal disease in Indigenous people [5]. Prostatic abscess resulting from *S. aureus* infection is very uncommon relative to its propensity to cause other human infections, with only 39 cases previously reported in the English language literature.

We have reported a case from our institution that had a common presentation of urinary retention as well as numerous risk factors identified in our literature search. These included diabetes mellitus, instrumentation of the urinary tract, and bladder outlet obstruction. Both the diagnosis of crusted scabies and a great toe ulcer are risk factors for bacteraemia and in the absence of chronicity, one of these percutaneous routes is likely to have been a source for his *S. aureus* bacteraemia and prostatic abscess.

The *S. aureus* isolates in this case were genotyped as CC5-MRSA and carried genes for the bi-component leukocidin PVL and was resistant to trimethoprim. This has similar genotypic properties to an emerging clone in the neighbouring jurisdiction of north Western Australia [11].

From a limited sample size, it appears that with prompt identification, diagnosis and management, this condition is treatable with a low case fatality rate. However, there are no guidelines for management of *S. aureus* prostatic abscess. Treatment of prostatic abscesses usually involves the use of broad-spectrum antibiotics directed at Enterobacteriaceae, with or without drainage. In our experience the decision to drain an abscess depends on several factors: the size of the abscess, accessibility and ease of drainage, and the patient’s response to antibiotic therapy. The sample size of this study and methodology involved means that we cannot draw firm conclusions on the evidential basis of draining prostatic abscesses caused by *S. aureus*. A single-centre retrospective study of drainage in prostatic abscess recommended that trans-rectal drainage is the best option for drainage in selected cases of prostatic abscess [12].

Our case is the first report of using clindamycin step-down therapy following IV therapy. Our use of clindamycin was driven by its excellent prostatic penetration, in which prostatic levels of clindamycin have been reported to exceed that of serum levels [13]. Options for drainage of prostatic abscess include trans-rectal, trans-perineal or transurethral approaches. In the case we reported here, the abscess was drained trans-rectally and was uncomplicated in this patient.

### Further research

There is variable consistency in what was reported in the literature and this made it difficult to synthesise the results to make recommendations for practice. Further research and reporting of *S. aureus* prostatic abscess with a standardised approach is required for understanding the pathogenesis and treatment of this uncommon condition. In this study, we have reported correlations between risk factors and the development of *S. aureus* prostatic abscess.

## Conclusions

Prostatic abscess is an uncommonly reported manifestation of *S. aureus* infection that has a non-specific clinical presentation, which can include abdominal and/or urinary symptoms. Most published cases have been due to MRSA. Specific risk factors include diabetes mellitus, immunosuppression, instrumentation of the urinary tract, and bladder outlet obstruction. Drainage is likely to accelerate cure but the optimal method remains undefined. While there are no published guidelines, clindamycin may be particularly useful for directed therapy of susceptible organisms given its excellent prostate penetration (along with bactericidal antibiotics in cases with bacteraemia).

## Additional files

Additional file 1: Figure S1. CT of pelvis showing prostatic abscess in the left lobe of the prostate. (PNG 477 kb)

Additional file 2: Figure S2. CT of pelvis showing persistence of prostatic abscess in the left lobe of the prostate. (PNG 447 kb)

## Abbreviations

BPH: Benign Prostatic Hypertrophy
CT: Computed Tomography
HIV: Human immunodeficiency virus
IDC: Indwelling catheter
IV: Intravenous
MRSA: Methicillin Resistant *Staphylococcus aureus*
MSSA: Methicillin Sensitive *Staphylococcus aureus*
nmMRSA: non–multidrug-resistant, methicillin-resistant *Staphylococcus aureus*
PVL: Panton-Valentine leucocidin
SA: *Staphylococcus aureus*
T2DM: Type 2 Diabetes mellitus
TMP-SMX: Trimethoprim/sulfamethoxazole
TURP: Transurethral Resection of the Prostate

## References

[1] Langer JE, Cornud F (2006). Inflammatory disorders of the prostate and the distal genital tract. Radiol Clin.

[2] Weinberger M, Cytron S, Servadio C, Block C, Rosenfeld JB, Pitlik SD (1988). Prostatic abscess in the antibiotic era. Rev Infect Dis.

[3] Morse LP, Moller C-CB, Harvey E, Ward L, Cheng AC, Carson PJ (2009). Prostatic abscess due to Burkholderia pseudomallei: 81 cases from a 19-year prospective Melioidosis study. J Urol.

[4] Ashdown LR (1979). An improved screening technique for isolation of pseudomonas pseudomallei from clinical specimens. Pathology [Phila].

[5] Tong SYC, Bishop EJ, Lilliebridge RA, Cheng AC, Spasova-Penkova Z, Holt DC (2009). Community-Associated Strains of Methicillin-Resistant *Staphylococcus aureus* and Methicillin-Susceptible *S. aureus* in Indigenous Northern Australia: Epidemiology and Outcomes. J Infect Dis.

[6] Lipsky BA, Byren I, Hoey CT (2010). Treatment of bacterial prostatitis. Clin Infect Dis.

[7] Lilliebridge RA, Tong SYC, Giffard PM, Holt DC (2011). The Utility of High-Resolution Melting Analysis of SNP Nucleated PCR Amplicons—An MLST Based *Staphylococcus aureus* Typing Scheme. PLOS ONE.

[8] Zheng X, Wang X, Zhou J, Xiang J, Xie L (2015). Diagnosis and treatment of community-associated methicillin-resistant Staphylococcus Aureus prostatic abscess involving the seminal vesicle: a case report. Exp Ther Med.

[9] Chia JK, Longfield RN, Cook DH, Flax BL (1986). Computed axial tomography in the early diagnosis of prostatic abscess. Am J Med.

[10] Liu C, Bayer A, Cosgrove SE, Daum RS, Fridkin SK, Gorwitz RJ (2011). Clinical practice guidelines by the Infectious Diseases Society of America for the treatment of methicillin-resistant Staphylococcus Aureus infections in adults and children. Clin Inf Dis.

[11] Coombs G. Western Australian methicillin-resistant *Staphylococcus aureus* [MRSA] epidemiology and typing report July 1 2014 to June 30 2015 [internet]. School of Veterinary and Life Sciences: Murdoch University; 2015 [cited 2017 Jul 20]. Available from: http://ww2.health.wa.gov.au/~/media/Files/Corporate/general%20documents/Infectious%20diseases/PDF/HISWA/accessannual-report-2015-16.ashx.

[12] Elshal AM, Abdelhalim A, Barakat TS, Shaaban AA, Nabeeh A, Ibrahiem E-H (2014). Prostatic abscess: objective assessment of the treatment approach in the absence of guidelines. Arab J Urol.

[13] Charalabopoulos K, Karachalios G, Baltogiannis D, Charalabopoulos A, Giannakopoulos X, Sofikitis N (2003). Penetration of antimicrobial agents into the prostate. Chemotherapy.

[14] Jana T, Machicado JD, Davogustto GE, Pan J-J (2014). Methicillin-resistant Staphylococcus Aureus prostatic abscess in a liver transplant recipient. Case Rep Transplant.

[15] Dubos M, Barraud O, Fedou A-L, Fredon F, Laurent F, Brakbi Y (2014). Prostatic abscesses and severe sepsis due to methicillin-susceptible Staphylococcus Aureus producing Panton-valentine leukocidin. BMC Infect Dis.

[16] Docekal J, Hall J, Reese B, Jones J, Ferguson T (2013). A rare presentation of community acquired methicillin resistant Staphylococcus Aureus. Case Rep Infect Dis..

[17] Lachant DJ, Apostolakos M, Pietropaoli A (2013). Methicillin resistant Staphylococcus Aureus prostatic abscess with bacteremia. Case Rep Infect Dis.

[18] Naboush A, Abou Yassine A, Yasmin M, Mobarakai N (2013). Community-acquired methicillin-resistant Staphylococcus Aureus prostatic abscess presenting as acute urinary retention: a case report and review of the literature. Case Rep Infect Dis..

[19] Krishnamohan P, Schaninger T, Baddour LM, Al-Hasan MN (2013). Community-acquired methicillin-resistant Staphylococcus Aureus prostatic abscesses. Am J Med Sci.

[20] Deshpande A, Haleblian G, Rapose A. Prostate abscess: MRSA spreading its influence into gram-negative territory: case report and literature review. BMJ Case Rep. 2013;2013 10.1136/bcr-2013-009057 PMC3618833 23531939

[21] Kiehl N, Kinsey S, Ramakrishnan V, Dajusta DG (2012). Pediatric prostatic abscess. Urology.

[22] Flannery MT, Humphrey D (2012). Case report of a prostatic abscess with a review of the literature. Case Rep Med.

[23] Dattilo WR, Shiber J (2013). Prostatitis or prostatic abscess. J Emerg Med.

[24] Park SC, Lee JW, Rim JS (2011). Prostatic abscess caused by community-acquired methicillin-resistant Staphylococcus Aureus. Int J Urol.

[25] Abreu D, Arroyo C, Suarez R, Campolo H, Izaguirre J, Decía R, et al. Community-acquired methicillin resistant Staphylococcus Aureus: a new aetiological agent of prostatic abscess. BMJ Case Rep. 2011;2011 10.1136/bcr.10.2010.3463 PMC3094799 22696740

[26] Chao BH, Kidd JM, Dow AW (2009). Methicillin-resistant Staphylococcus Aureus bacteremia due to prostatic abscess. J Hosp Med.

[27] Baradkar VP, Mathur M, Kumar S (2008). Prostatic abscess by Staphylococcus Aureus in a diabetic patient. Indian J Med Microbiol.

[28] Gautam M, Gandhi A, Rose F (2008). Methicillin-resistant *Staphylococcus aureus*: fatal prostatic abscess in an AIDS patient. South Med J.

[29] Pierce JR, Saeed Q, Davis WR (2008). Prostatic abscess due to community-acquired methicillin-resistant Staphylococcus Aureus. Am J Med Sci.

[30] Lin MY, Rezai K, Schwartz DN (2008). Septic pulmonary emboli and bacteremia associated with deep tissue infections caused by community-acquired methicillin-resistant Staphylococcus Aureus. J Clin Microbiol.

[31] Tobian AAR, Ober SK (2007). Dual perinephric and prostatic abscesses from methacillin-resistant Staphylococcus Aureus. South Med J.

[32] Shindel AW, Darcy MD, Brandes SB (2006). Management of prostatic abscess with community-acquired methicillin-resistant Staphylococcus Aureus after straddle injury to the urethra. J Trauma.

[33] Baker SD, Horger DC, Keane TE (2004). Community-acquired methicillin-resistant Staphylococcus Aureus prostatic abscess. Urology.

[34] Fraser TG, Smith ND, Noskin GA (2003). Persistent methicillin-resistant Staphylococcus Aureus bacteremia due to a prostatic abscess. Scand J Infect Dis.

[35] Savarirayan S, Shenykin Y, Gerard P, Wise GJ (1995). Staphylococcus periprostatic abscess: an unusual cause of acute urinary retention. Urology.

[36] Trauzzi SJ, Kay CJ, Kaufman DG, Lowe FC (1994). Management of prostatic abscess in patients with human immunodeficiency syndrome. Urology.

[37] Gill SK, Gilson RJ, Rickards D (1991). Multiple prostatic abscesses presenting with urethral discharge. Genitourin Med.

[38] Zagoria RJ, Papanicolaou N, Pfister RC, Stafford SA, Young HH (1987). Seminal vesicle abscess after vasectomy: evaluation by transrectal sonography and CT. AJR Am J Roentgenol.

[39] Beckman TJ, Edson RS (2007). Methicillin-resistant *Staphylococcus aureus* prostatitis. Urology.

[40] Javeed I, Kaushik P, Chowdhury M, Palermo B, Emery CL (2012). Community acquired methicillin resistant *Staphylococcus aureus* [CA-MRSA] prostatic abscess in a diabetic patient. Int J Case Rep Images.

[41] Sheahan G, Vega Vega A (2013). Dramatic complications of prostatis: a prostatic abscess and scrotal abscess. ANZ J Surg.

[42] Sukhal S, Zamora JG, Herrera P (2013). An unusual cause of prostatic abscess: a case report and review of literature. Infect Dis Clin Pract.

[43] Nargund VH, Stewart PAH (1995). Acute bacterial prostatitis with osteomyelitis. J R Soc Med.

[44] Shokeir AA, Dawaba M, Abdel-Gawad M, El-Azab M, Shokeir MA (1995). Prostatic abscess in a child. Scand J Urol Nephrol.

[45] Hoffman MA, Steele G, Yalla S (2000). Acute bacterial endocarditis secondary to prostatic abscess. J Urol.

[46] Mann S (1960). Prostatic abscess in the newborn. Arch Dis Child.

