# Supplementary figures and images for: Staphylococcus aureus Prostatic abscess: a clinical case report and a review of the literature
Source: BMC Infect Dis. 2017 Jul 21;17:509. doi: 10.1186/s12879-017-2605-4 (PMC5521102; doi:10.1186/s12879-017-2605-4)

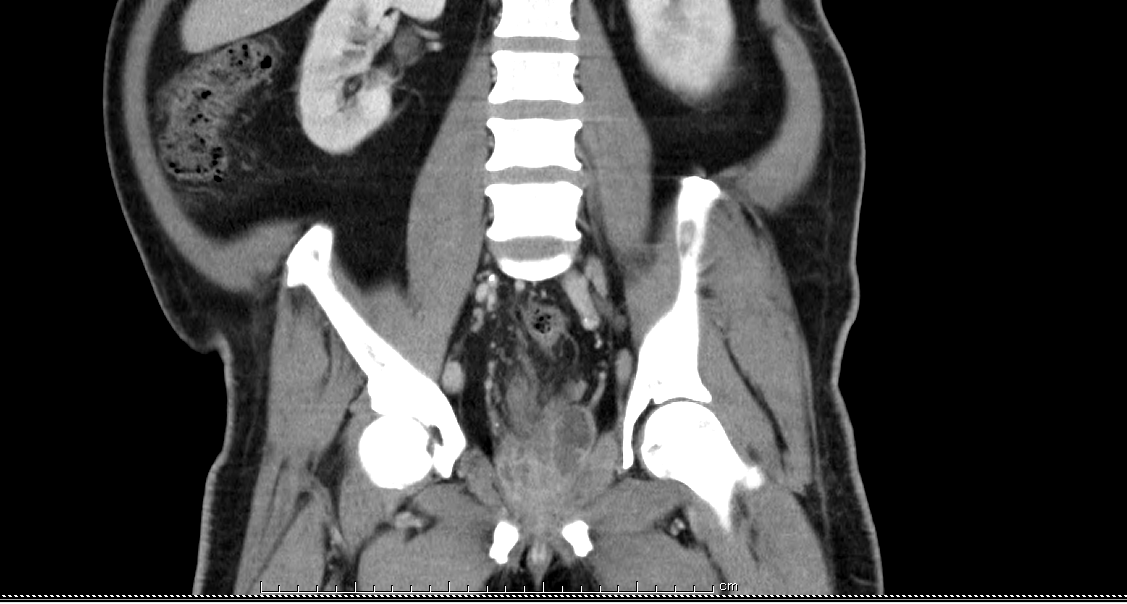

Supplement: Supplementary file 1 — CT of pelvis showing prostatic abscess in the left lobe of the prostate. (PNG 477 kb) [file 12879_2017_2605_MOESM1_ESM.png]

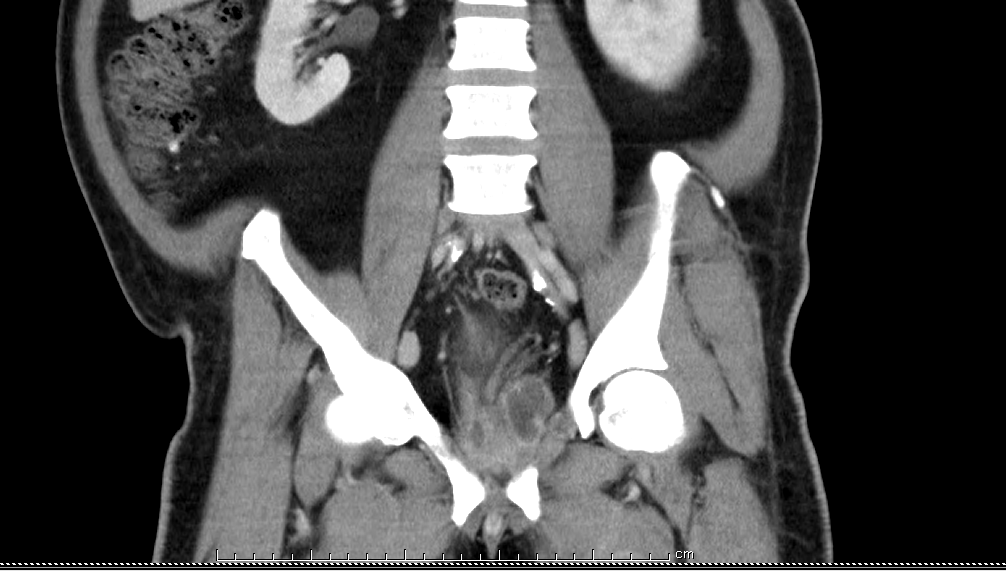

Supplement: Supplementary file 2 — CT of pelvis showing persistence of prostatic abscess in the left lobe of the prostate. (PNG 447 kb) [file 12879_2017_2605_MOESM2_ESM.png]
